# Massive hemoptysis as a sole presentation of left atrial myxoma

**DOI:** 10.1093/jscr/rjad301

**Published:** 2023-05-29

**Authors:** Narmeen Giacaman, Nizar Marzouqa, Elias Saeed, Salem M Tos, Ahmad Emar, Mohammad Nassr, Bajis Amr, Bisher Marzouqa

**Affiliations:** Department of Internal Medicine, Al-Quds University, Jerusalem, State of Palestine; Department of Internal Medicine, Al-Quds University, Jerusalem, State of Palestine; Department of Internal Medicine, Al-Quds University, Jerusalem, State of Palestine; Department of Internal Medicine, Al-Quds University, Jerusalem, State of Palestine; Cardiology Department, Al-Ahli Hospital, Hebron, State of Palestine; Cardiology Department, Al-Ahli Hospital, Hebron, State of Palestine; Cardiology Department, Al-Ahli Hospital, Hebron, State of Palestine; Cardiovascular Surgery Unit, Al-Ahli Hospital, Hebron, State of Palestine

**Keywords:** Hemoptysis, Cardiac Mass, Left atrium, Myxoma

## Abstract

Diagnosis of myxoma can be difficult given its variable presentation and while adopting common sense in diagnosing this condition, physicians should also be aware of atypical presentations. Herein we present a 47-year-old heavy smoker presented with massive blood-stained expectoration. He was later diagnosed with cardiac myxoma and managed accordingly.

## INTRODUCTION

Hemoptysis is considered a diagnostic challenge with a differential diagnosis that encompasses a wide range of potential etiologies. In this presented case, the first presentation of cardiac myxoma was massive hemoptysis and Physical examination had a main role in reaching the diagnosis in a timely manner. In this case, we would like to highlight this rare presentation of left cardiac myxoma and emphasize the importance of history taking and physical examination, though they are basic routines, they are still the cornerstone in carrying out on-target diagnostic efforts.

## CASE REPORT

A 47-year-old male patient, a heavy smoker, presented to our hospital with massive blood-stained expectoration of ⁓250 ml/day for the last 2 days. On presentation, the Patient did not complain of any other symptoms, including chest pain, upper or lower respiratory infection symptoms, palpitations, fatigue, lightheadedness and shortness of breath. A few days preceding the onset of hemoptysis, he had flu-like symptoms. The patient denied any history of urine discoloration, weight loss, chronic cough or arthralgia. The patient has a free past medical and surgical history. He doesn’t routinely take any medications. Vital Signs were within normal limits at presentation.

Upon physical examination, he had no significant findings, except for a loud S1 and bilateral fingers clubbing.

Following a thorough history and physical examination, basic laboratory blood tests were ordered and results were within normal limits except for a low Hemoglobin of 12 g/dl.

**Figure 1:**
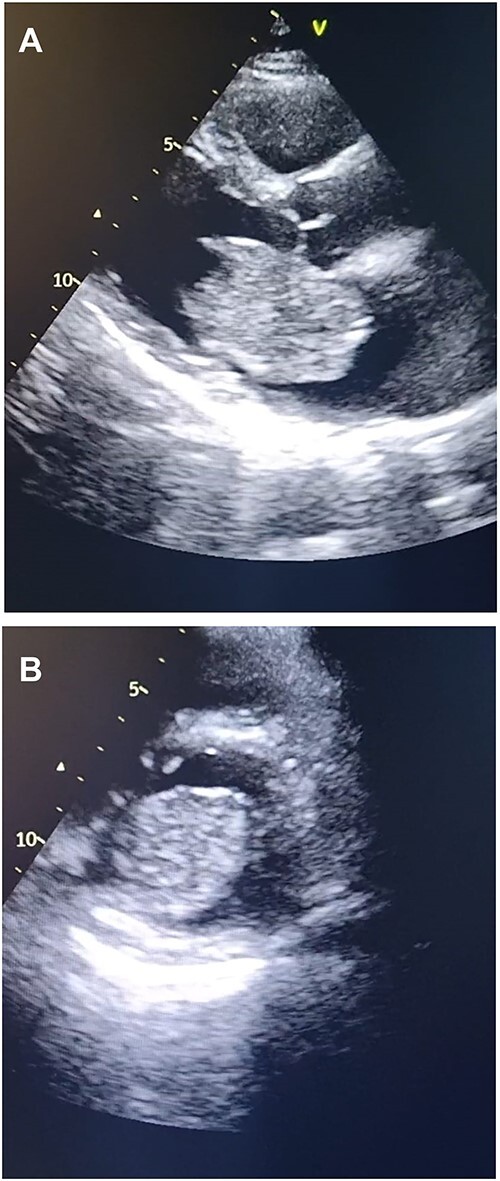
Transthoracic echocardiography. (**A**) Echocardiogram parasternal long-axis view (PLAX) view showing large homogenous mobile mass in LA. (**B**) Echocardiogram parasternal short-axis view (PSAX) view showing Left atrial mass protrusion through the Mitral valve during diastole.

**Figure 2:**
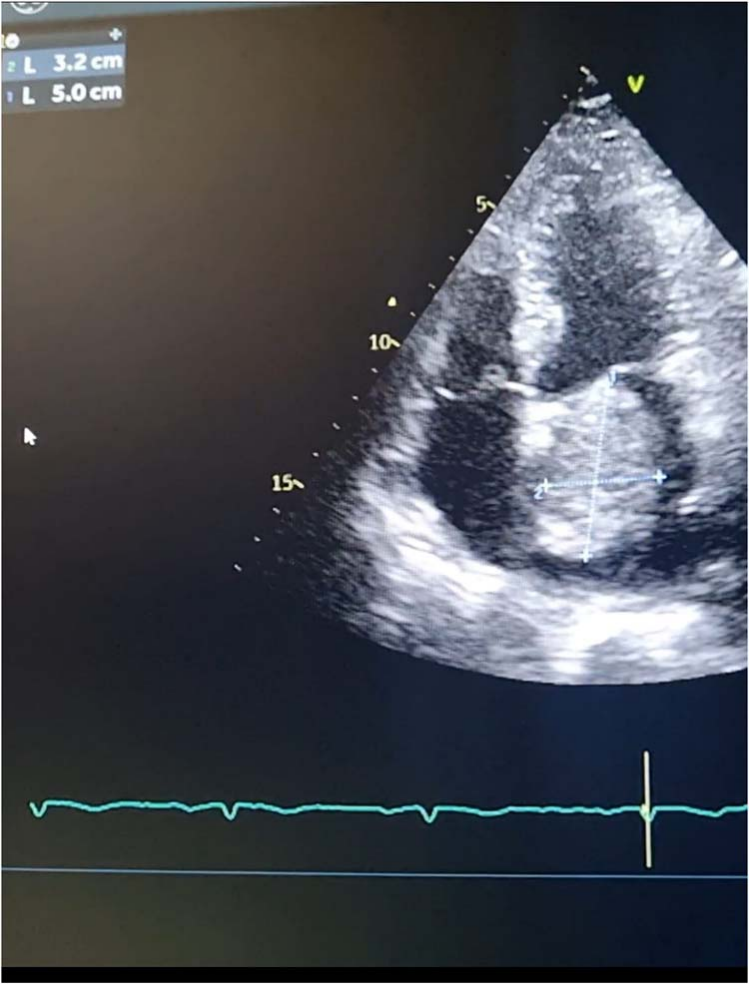
Transthoracic echocardiography. 2D measurements of the LA mass measuring 5 cm ^*^ 3.2 cm.

Meanwhile, transthoracic echocardiography was done to investigate the cause of loud S1 and fingers clubbing. The Echocardiography showed a large heterogeneous mass attached to the fossa ovalis in the left atrium. (Figs 1A, B and 2). This was followed by obtaining a computed tomography (CT) scan of the chest to investigate for other causes of hemoptysis. The CT scan showed no other identifiable etiology precipitating hemoptysis. Thus, it was determined that elevated pressure in the pulmonary vessels secondary to obstruction in the mitral valve blood flow caused hemoptysis in the presented case. A preoperative diagnostic coronary angiography showed normal coronaries with no evidence of stenosis.

**Figure 3:**
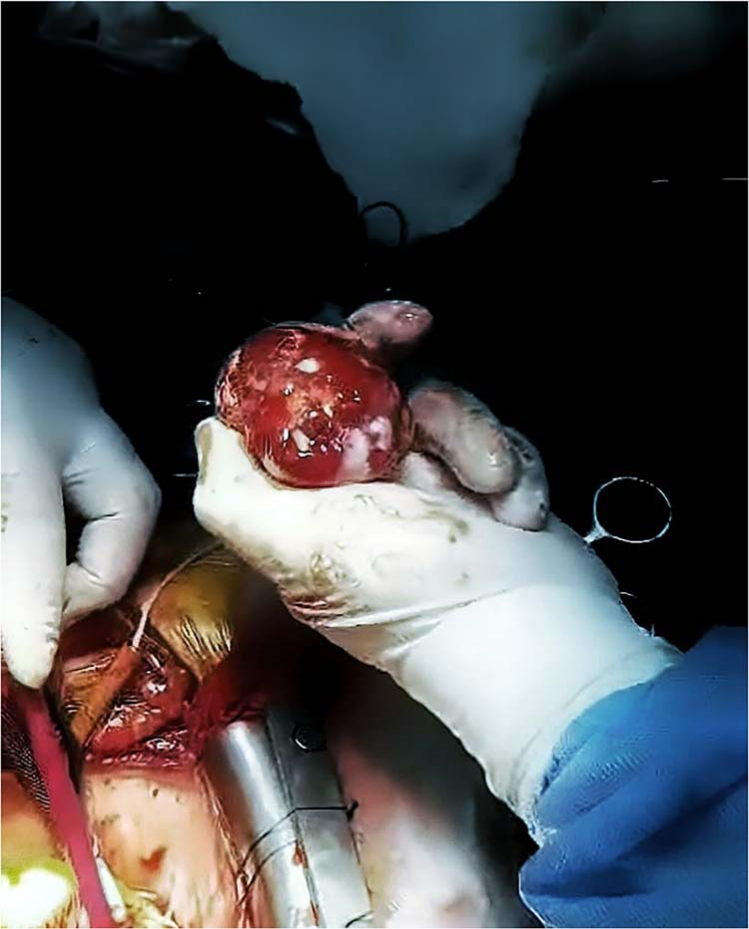
The LA mass after excision. The heterogeneous mass after being excised during open heart surgery.

Open heart surgery was performed and surgical excision of the left atrial mass measuring 5^*^4.7^*^3.5 cm (Fig. 3) with an exploration of the heart chambers was performed. In addition to the excision of a part of the interatrial septum. The mass was sent for cellular and pathologic evaluation that concluded the diagnosis of myxoma (myxoma cells positive for calretinin immunostain) and was negative for malignancy.

On follow up the patient reported complete clinical recovery without any signs or symptoms.

## DISCUSSION

Primary cardiac tumors are rare and mostly benign. The majority of benign cardiac tumors in adults are myxomas [1–4, 6, 7]. Myxomas often arise from the left atrium, specifically, the interatrial septum opposite the Fossa Ovalis. Additionally, they occur more commonly in women [1, 2, 6]. The presentation of left atrial myxomas varies widely, from asymptomatic [3] to lethal [1]. Despite being ‘benign’ in terms of malignancy potential, they carry life-threatening complications due to their ability to ‘embolize’ [1, 3, 6, 7]. The most frequent signs and symptoms can be summarized in a triad of cardiac-obstructive, embolic and constitutional symptoms [1, 2, 5, 7].

Myxoma-related hemoptysis is mentioned in literature in different contexts: as a sole first symptom [4], alongside other symptoms [5], or as a part of a coexisting disease, like bronchiectasis [3]. The exact incidence of hemoptysis isn’t known. However, hemoptysis seems to be recorded more with right-sided myxomas, as evidenced by the existence of fewer published left-sided cases with this symptom [4]. One series of 112 patients with left cardiac myxomas only had three patients with ‘abdominal pain or hemoptysis’ [2]. What was unique in our case is the fact that our patient had hemoptysis without any other complaints. Only a few similar reports exist [3, 4]. This usually constitutes a diagnostic challenge due to the wide range of possible differential causes.

No specific blood test or marker has been directly associated with myxoma, yet anemia (as in our patient), high C-reactive protein (CRP) and high Erythrocyte sedimentation rate (ESR) have been previously documented [1]. Physical examination can vary a lot as well, depending on the site, size and mobility of the tumor. For example, auscultation may reveal a loud S1, as seen in our patient, or a ‘tumor plop’, a low-pitched early diastolic sound, that aids in differentiating left atrial myxoma from other cardiac diseases [1, 7]. Moreover, literature has shown that clubbing, another sign in our case, is also a known possible finding [1]. Echocardiography remains the most appropriate test for myxomas, with Transesophageal Echocardiography having a slight advantage over the transthoracic approach [1, 5]. Immunohistochemical testing of the tumor may help confirm the diagnosis. Calretinin, the test we used, identified up to 100% of cardiac myxomas in some studies [1, 6].

The mainstay treatment is surgical resection as soon as possible, to avoid serious embolic sequelae. Different approaches are described in the literature and there are several mentions of partial interatrial septum excision [1, 2, 7]. The post-surgical and long-term prognoses are excellent, with low postoperative mortality and recurrence rates [1, 2, 5–7].

In Conclusion, this case highlighted a rare presentation of left atrial myxoma as massive hemoptysis in an adult male. Diagnosis of left atrial myxoma can be challenging due to its variable presentation and absence of specific diagnostic markers. This challenge is further compounded when the presentation is atypical and singled out by an unusually attributed symptom. Clinicians should be aware of this rare entity and include it in the differential diagnosis of unexplained hemoptysis, particularly in the presence of a loud S1 or other suggestive cardiac symptoms.

## Data Availability

The data sets generated and analyzed during the current study are available from the guarantor Doctor Ahmad Emar on reasonable request.
